# Ear injury as the only manifestation of amyloidosis

**DOI:** 10.5935/1808-8694.20130020

**Published:** 2015-10-14

**Authors:** Catia Rodrigues Domingos, Rubiana Ferreira Sousa, Helena Maria Gonçalves Becker, Paulo Fernando Tormin Borges Crosara, Roberto Eustáquio Santos Guimarães

**Affiliations:** MD; MSc in Sciences. Otorhinolaryngologist and Head and Neck Surgeon - Hospital São José e Centro Hospitalar Unimed - Joinville - SC; MD. Chest Surgeon - Hospital São José e Centro Hospitalar Unimed - Joinville - SC; MD; Head and Neck Surgeon and Maxillo-Facial-Skull Surgeon -SC; Hospital das Clínicas da UFMG

**Keywords:** amyloidosis, external ear

## INTRODUCTION

Amyloidosis is a disease of deposits classified into systemic and localized^1^. The head and neck involvement is rare, and the larynx is the most affected site^2^, ^3^. Ear amyloidosis is a rare occurence^4^. The definitive diagnosis is histopathological^1^. The disease is more frequent between 50-70 years of age, and it predominates in men (3:1). Its etiology is unknown^1^.

Because of the severity of the systemic form of the disease and its association with plasmocytoma and multiple myeloma, it is important to distinguish these manifestations^5^.

We present here a case of a 42-year-old patient with ear amyloidosis.

## CASE PRESENTATION

**Figure 1:**
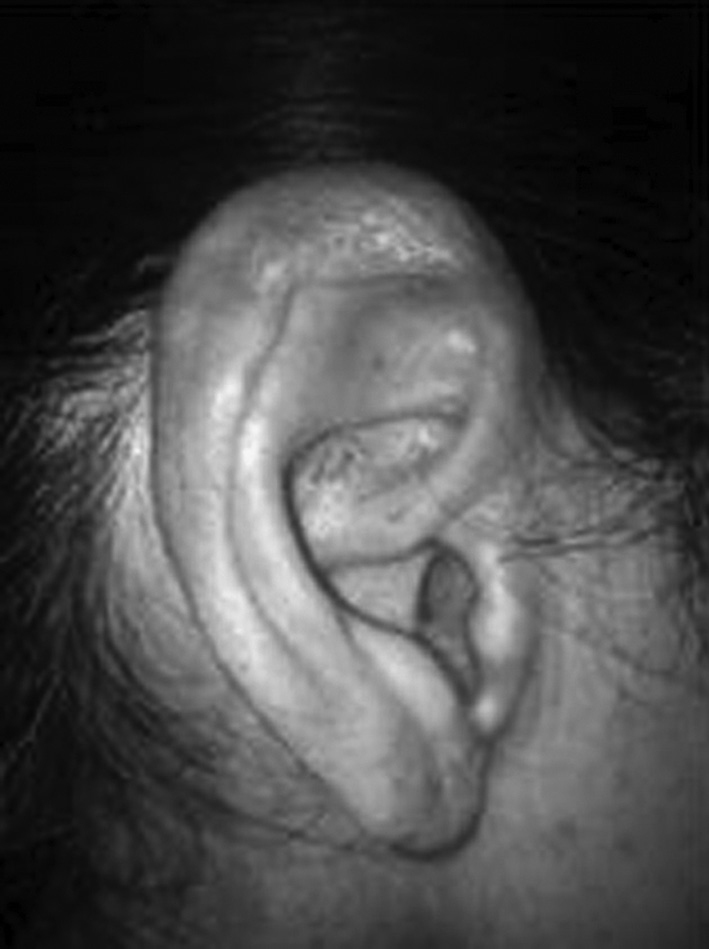
Amyloidosis lesion on the ear pinna.

M.A.S., 42 years old, female, referred to us with recurrent otalgia and hearing loss secondary to amyloidosis, diagnosed 21 years ago, she reported onset of earache and fullness in the ear for about 4 days before coming to the clinic, besides floating hearing loss. She was using amitriptyline, Arcox^®^ and codeine. Upon the exam, there were hyperemia and edema of the outer ear and pinna (Figure 1).

Otoscopy was difficult, because of edema in the canal, showing a normal ear drum.

The pathology exam of the pinna skin biopsy showed homogeneous eosinophilic material in the dermis and a positive dye for amyloidosis, with methyl violet.

We ordered lab exams: ESR, CBC, renal and liver function tests, beta 2 microglo-bulin, protein electrophoresis and abdominal subcutaneous tissue biopsy. They came back all normal. The pathology report from the subcutaneous tissue read: Normal fibrous-fat tissue and a search for amyloidosis with the Congo red dye with negative birefringent polarized light.

Vocal and tonal audiometry showed moderate hearing loss and tympanometric type A curve, bilaterally, with preserved stapes reflexes.

The external auditory canals and pinna were filled with topical steroid (0.5% clobetasone cream), once a week, for 4 weeks, evolving with symptom improvements.

Today, the patient is being observed.

## DISCUSSION

Amyloidosis is a rare disease, with deposits of protein fibrils. Such protein build up in the tissues may compromise the function of organs, such as the heart^1^. In 20%, there is multiple myeloma associated -plasmacytic neoplasia, medullary infiltration by plasmacytes, associated with the serum M protein (monoclonal immunoglobulin), and organic injury^5^.

In head and neck amyloidosis, systemic involvement must be ruled out by laboratory tests, such as protein electrophoresis, renal function test, electrocardiogram and abdominal ultrasound^2^. The study of monoclonal production of immunoglobulins is important^4^; the systemic form of the disease happens when there is monoclonal protein in the serum or urine and organ involvement, such as albuminuria (amyloidosis corresponds to 10% of the non-diabetic nephrotic syndromes in adults), heart disease, hepatomegaly, neuropathy and bone marrow infiltrated by at least 10% of plasmocytes^4^^,^^5^.

In this paper, we report partial and temporary stenosis of the external auditory canal, affecting the pinna - a rare manifestation of amyloidosis. In the few reports present in the literature, fullness of the ear and hearing loss are constant findings, which did not happen with pain - which was not reported by other papers.

In most cases, amyloidosis is systemic and follows multiple myeloma^5^. The treatment proposed for amyloidosis in the literature is based on lesion exeresis^4^.

## CONCLUSION

There are but a few studies on amyloidosis. Among the localized forms, the ear form is rare. The definition of clinical and laboratory characteristics is important for its diagnosis. It is important to investigate multiple myeloma in the systemic form of the disease.
